# Intestinal Obstruction

**Published:** 1907-01

**Authors:** 


### INTESTINAL OBSTRUCTION.

Intestinal obstruction as a post-operative complication is the division of the subject to which the attention of the abdominal surgeon is sometimes called with great force, and in cases in which he least expects it is a complication The question that naturally arises in the mind of the operator is, what are the predisposing causes, and what are the conditions in the abdomen immediately following an operation, that are direct causative factors? Predisposing causes may be divided into those which are inherent in the patient herself and those which are produced artificially at the time of operation and immediately subsequent thereto. The predisposing causes in the patient herself are found in generally poorly nourished individuals with asthenia, and a tendency towards chronic constipation. Predisposing causes at the time of operation are over-handling of the intestine or other abdominal viscera, exposure of the abdominal contents to the air, a prolonged operative procedure.

The conditions subsequent to an operation are the administration of any drug which tends to paralyze the circular muscular fibers of the bowel, and the over use of cathartics.

The diagnosis is often difficult and sometimes almost impossible during the period of time in which an operation is of greatest value. The general appearance of the patient is a very great help, the face is drawn, the patient is anxious, later they become apathetic, the pulse, as a rule, increases in rapidity but it may not be over one hundred. The temperature does not rise at first and usually not until the toxemia is well developed. Vomiting is not always an early symptom, especially if the obstruction is low down in the small gut; when it does occur it is first the contents of the stomach, later bile and last fecal. Pain may or may not be present and is not a symptom on which much dependence can be placed.

Distention of the abdomen is not a reliable symptom. Complete obstruction may be present and there may be ver ylittle distention until late. The fact that no gas has passed and no fecal matter except that which is contained in the lower bowel are points that are of very great value; but it is not to be forgotten that where frequent and copious enemas have been given, the patient may pass the air that has been carried in with the enema in such a manner as to be delusive. There is one symptom to which too great importance cannot be given, and that is the peculiar boggy feeling of the gut above the obstruction. Where this peculiar bogginess is felt in the abdomen, a diagnosis of intestinal obstruction may be made with surety, and it is the writer's opinion that this is so characteristic that an operation is justifiable even if the other symptoms are uncertain; that is, if, after an operation, no gas or fecal matter has been expelled and there is an area to be felt in the abdomen of this peculiar, inelastic character; even if there has been no vomiting, the pulse and temperature are not alarming, the diagnosis is almost certain, and the time for operation has come, later, when the symptoms become more classical, the chances of relief by operation are very much less.

The treatment of this condition divides itself into preventitive and operative. Preventitive treatment is perhaps the best and most logical. In patients with a poorly nourished asthenic body, abdominal operations should be avoided if possible; if not, every precaution should be used both prior to, at the time of, and subsequent to the operation; that is, the patient's general condition should be improved as much as possible. At the time of operation no undue risks should be taken, and special care should be had after operation.

The diet and care of a patient subsequent to and after operation are important factors in obtaining good results but cannot be considered here. There are several points, however, at the time of operation which can be summed up in a few words, namely: The time of operation should be as short as is consistent with careful surgery. The exposure of the intestines and omentum to the air should be avoided, the abdominal pads should be moistened in sterile hot salt solution, and the part of the abdomen that is not to be interfered with should be well walled off, the peritoneum should not be handled more than is unavoidable. Raw surfaces, if it is possible, should be covered by peritoneal flaps. It is the writer's opinion that the peritoneum is not damaged so much by smooth rubber gloves as it is by the naked hand.

The use of morphine after abdominal section while frequently necessary is not without danger; first, in causing intestinal stasis; second, in masking symptoms. When given it should be accompanied by strychnine. The use of cathartics where acute intestinal obstruction exists, cannot be too strongly condemned, as it only increases the distention above the obstruction and can do no possible good.

Where a diagnosis of intestinal obstruction is made, operation is the only rational treatment and it is not to be forgotten that even a a few hours' delay may be the cause of a fatal termination.—*Ed. Annals Gyn. and Pediat.*, November, 1906.

